# *Anaplasma platys* beyond canines: a systematic review of host range, phylogenetic relatedness, and knowledge gaps in Africa

**DOI:** 10.1186/s13071-026-07278-4

**Published:** 2026-02-18

**Authors:** Zamantungwa Thobeka Happiness Mnisi, Sekgota Marcus Makgabo, Charles Byaruhanga

**Affiliations:** 1 DSTI-NRF SARChI Chair (Ecosystem Health), Department of Biodiversity, University of Limpopo, Private Bag X1106, Sovenga, 0727 South Africa; 2 Department of Veterinary Tropical Diseases, Faculty of Veterinary Science, University of Pretoria, Onderstepoort, South Africa; 3 Vaccine and Diagnostic Development Programme, Onderstepoort Veterinary Research, Agricultural Research Council, Pretoria, South Africa

**Keywords:** *Anaplasma platys*, *Anaplasma platys*-like, Africa, *Rhipicephalus sanguineus* sensu lato, Host, Phylogenetic, Systematic review

## Abstract

**Background:**

*Anaplasma platys* is a causative agent of canine cyclic thrombocytopenia, transmitted by *Rhipicephalus sanguineus* sensu lato. Reports of *A. platys* in Africa remain scarce and fragmented, with most detections occurring as co-infections in broader *Anaplasmataceae* surveys.

**Methods:**

A systematic review was conducted from February to May 2025, analyzing all peer-reviewed journal articles, theses, and conference proceedings published in English in three databases—PubMed, Web of Science, and Scopus—from database inception up to and including December 2024. Following screening, 103 full-text peer-reviewed records were deemed eligible for data extraction. The outcome of interest was *A. platys* and *A. platys*-like detection by various methods and the corresponding sequences (16S ribosomal RNA (rRNA) and *groEL* genes) from GenBank for use in phylogenetic analyses.

**Results:**

*Anaplasma platys* and *A. platys*-like were detected in 80 studies in 25 of the 54 African countries across multiple host species, and there was no detection in the four semi-autonomous or autonomous territories. The pathogen was mostly detected in domestic dogs, with prevalence that ranged from 0.8% to 100%, followed by cattle, with prevalence of 0.2–84%, and sheep with 1.7–100%. Other domestic animals included goats (6.7–55.7%) and camels (0.7–61.1%), while wildlife included impala (9.5–58.3%), African buffalo (3.6–7.7%), sable antelope (4.3%), Grant’s gazelle (32.4%), kudu (83.3%), zebra (16.7%), warthog (12.5%), elephant (50%), lion (16.7%), leopard (11.1%), bat-eared fox (88.9%), brown hyena (82.3%), and spotted hyena (100%). Additionally, *A. platys* DNA was detected in ticks, mainly *R. sanguineus* s.l. but also *Rhipicephalus pulchellus*, *R. annulatus*, *R. pravus*, *R. evertsi evertsi*, *R. microplus*, *R. simus*, *R. humeralis*, *R. camicasi*, *Haemaphysalis leachi*, and *Hyalomma excavatum*, as well as in fleas (*Pulex irritans*, *Ctenocephalides felis felis*, and *Ctenocephalides felis canis*). One documented human case involved a veterinarian who had traveled to South Africa, raising concerns about zoonotic potential, though the infection source remains unclear. The 16S rRNA phylogenetic tree demonstrated broad host and vector diversity, while the *groEL*-based analysis resolved distinct bovine- and canine-associated lineages.

**Conclusions:**

These findings highlight a likely broad vertebrate host range of *A. platys* and possible association with multiple tick vectors. Critical knowledge gaps remain regarding host-specific genotypes and the role of tick species in transmission.

**Graphical Abstract:**

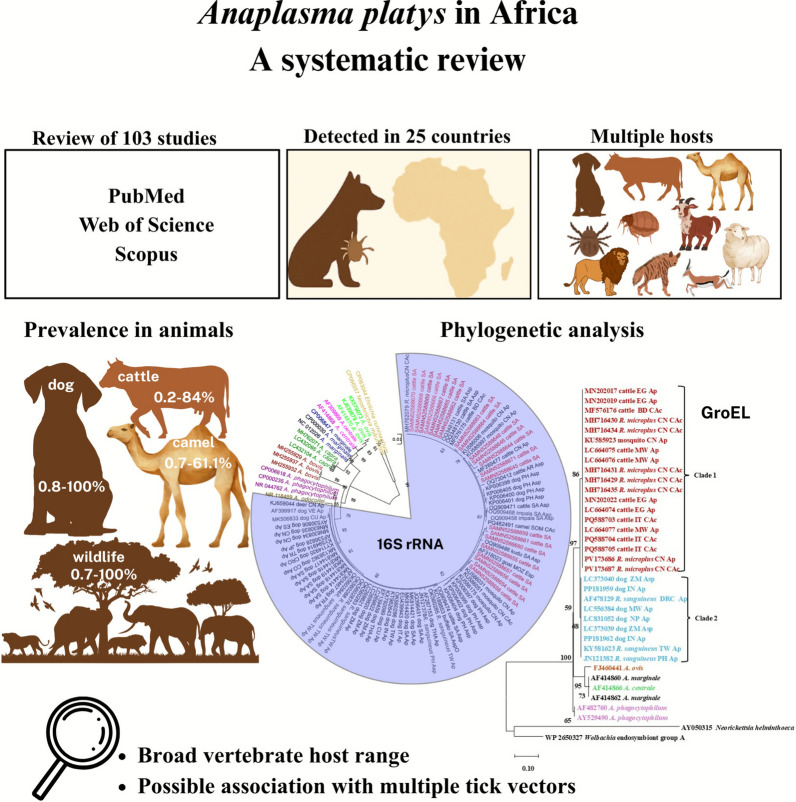

## Background

The earliest description of *Anaplasma platys* dates back to 1978, when Harvey and colleagues identified a *Rickettsia*-like organism infecting the platelets of dogs in Florida, United States of America and inducing cyclic episodes of thrombocytopenia [1]. The infection was marked by recurrent rickettsiaemia, with morulae observed within platelets and periodic declines in platelet counts occurring at 1- to 2-week intervals. Building on this discovery, the establishment of an indirect fluorescent antibody (IFA) test provided the first serological means of diagnosing infectious canine cyclic thrombocytopenia [2]. At the time, the organism was designated as *Ehrlichia platys* based on its morphology and cross-reactivity with *E. canis*. However, its classification was later transformed with advances in molecular phylogenetics. Comparative analyses of the 16S ribosomal RNA (rRNA) gene and *groESL* operon [3] demonstrated that the pathogen was more closely related to *Anaplasma phagocytophilum* and *Anaplasma marginale* than to *Ehrlichia* species. This finding led to the reorganization of genera within the families *Rickettsiaceae* and *Anaplasmataceae* and further reclassification of *E. platys* as *A. platys*. This taxonomic revision not only clarified the evolutionary position of the species but also highlighted its importance in the broader context of *Anaplasmataceae* biology.

Although primarily considered a canine pathogen, cases of *A. platys* infection or pathogen DNA have been detected worldwide in a range of mammalian hosts, including cats [4], foxes [5], wild boars [6], red deer [7], camels [8], goats, sheep [9], and cattle [10–12]. Ticks in the *Rhipicephalus sanguineus* complex are recognized as the primary vectors [13–16]. *Anaplasma platys* DNA has also been detected in several other tick species including *R. pulchellus*, *R. annulatus*, *R. pravus*, *R. evertsi evertsi*, *R. microplus*, *R. simus*, *R. humeralis*, *Haemaphysalis leachi*, and *Hyalomma excavatum* [17–21], as well as fleas collected from humans (*Pulex irritans*), cats (*Ctenocephalides felis felis*), and dogs (*C. felis canis*) [22]. Vertical transmission in dogs [23] and by transfusion of infected blood [1] have also been demonstrated. *Anaplasma platys* is classified within the “zoonotic” clade of the genus *Anaplasma* [24]. Its zoonotic potential is further supported by reports of human infections, including cases documented in the USA [1], South Africa [25], and Venezuela [26].

Despite its global distribution and zoonotic potential, reports of *A. platys* and *A. platys*-like organisms in Africa remain scarce and fragmented [17]. Nevertheless, both molecular and serological evidence indicate its presence across multiple African countries, including Kenya, Côte d'Ivoire, Gabon, Nigeria [8, 10], Angola, Sudan, Tunisia, Algeria, Morocco [27–32], and South Africa [12, 25, 33].

Although cattle are not considered typical hosts of *A. platys*, an increasing number of studies have reported detection of *A. platys* and *A. platys*-like DNA in cattle across Africa, making them the most frequently reported non-canine hosts, second only to dogs. Particularly high prevalence has been observed in Mozambique [34] and South Africa [12, 33]. Other countries with reports of detection include Nigeria [8, 10, 35], Senegal [36], Tunisia [37], and Uganda [38], highlighting the widespread presence of *A. platys* DNA in non-canine hosts and its potential implications for livestock health.

Despite increasing reports of *A. platys* and *A. platys*-like organisms in Africa, major gaps remain in understanding their prevalence, geographical distribution, and ecological determinants, as well as zoonotic potential. The circulating organisms detected across various hosts may represent true *A. platys* strains or distinct species, but this distinction is unresolved. Phylogenetic classification of *A. platys* remains unclear; a previous study showed that the pathogen is grouped with *Anaplasma* sp. (Omatjenne), *Anaplasma* sp. Mymensingh, and another unrecognized *Anaplasma* species [39]. Similarly, little is known about its biological transmission cycle, reservoir hosts, and antigenic properties, or whether arthropods beyond ticks contribute to its spread. The role of co-infections with other *Anaplasma* species in driving genetic recombination and novel variant emergence also remains unexplored, with potential implications for disease severity. Addressing these uncertainties, particularly in regions of high tick density, is critical to understanding the epidemiology of the pathogen, which is essential for improvement in diagnostics, control, and prevention strategies that are relevant to both animal and human health.

The aim of this systematic review is therefore to assess the occurrence of *A. platys* and *A. platys*-like organisms in a range of wild and domestic animals and humans as well as potential vectors in Africa, and to establish the 16S rRNA and *groEL* phylogenetic relatedness within *A. platys* strains and across *Anaplasma* species.

## Methods

### Study design and search strategy

This systematic review followed the Preferred Reporting Items for Systematic reviews and Meta-Analyses (PRISMA) guidelines [40]. It was not registered with any registry. Africa, for the purpose of this systematic review, encompasses 54 sovereign nations (United Nations Worldometer: https://www.worldometers.info/geography/how-many-countries-in-africa/), three semi-autonomous or autonomous territories, and one disputed dependent territory (with the exclusion of three European-dependent territories), and is geographically categorized into five regions (northern, eastern, western, southern, and central). The semi-autonomous territory is the island of Zanzibar (for Tanzania), and the autonomous states are Somaliland and Puntland (for Somalia). The three European-dependent territories that were excluded are Réunion (France), Mayotte (France), and Saint Helena (United Kingdom), and the included one is the Western Sahara, which is the disputed non-self-governing territory claimed by Morocco. The continent covers a total land area of 29,648,481 km^2^ and has a human population of over 1.52 billion as of September 9, 2024, representing 18.6% of the world’s total population (https://www.worldometers.info/world-population/africa-population/).

We searched and reviewed relevant literature regarding the occurrence and distribution of *A. platys* in vertebrate and invertebrate hosts in all African countries and regions. The search was conducted on Scopus, Web of Science, and PubMed from February through May 2025 and included all relevant publications from database inception up to and including December 2024. No time restrictions were applied to allow for a comprehensive review. The search string comprised two search topics, the pathogen and study area, and each comprised relevant search terms. The search topics were separated by the Boolean operator “AND,” while the search terms were separated by the Boolean “OR,” as follows: [(*Anaplasma platys* OR *A. platys* OR *Anaplasma platys*-like OR *A. platys*-like OR *Anaplasma platys* like OR *A. platys* like OR *Ehrlichia platys* OR *Ehrlichia platys*-like OR *E. platys* OR *E. platys*-like OR *E. platys* like) AND (Africa OR Algeria OR Angola OR Benin OR Botswana OR Burkina Faso OR Burundi OR Cameroon OR Cabo Verde OR Cape Verde or Republic of Cabo Verde OR Central African Republic OR Chad OR Comoros OR Congo OR Republic of the Congo OR Congo-Brazzaville OR Congo Republic OR DR Congo OR Democratic Republic of Congo OR Zaire OR Côte d’Ivoire OR Ivory Coast OR Djibouti OR Equatorial Guinea OR Egypt OR Eritrea OR Ethiopia OR Gabon OR Gambia OR Ghana OR Guinea OR Guinea-Bissau OR Kenya OR Lesotho OR Liberia OR Libya OR Madagascar OR Malawi OR Mali OR Mauritania OR Mauritius OR Morocco OR Mozambique OR Namibia OR Niger OR Nigeria OR Rwanda OR Sao Tome and Principe OR Sâo Tomé and Príncipe OR Senegal OR Seychelles OR Sierra Leone OR Somalia OR Somaliland OR Puntland OR South Africa OR South Sudan OR Sudan OR Swaziland OR Eswatini OR Tanzania OR United Republic of Tanzania OR Zanzibar OR Togo OR Tunisia OR Uganda OR Western Sahara OR Zambia OR Zimbabwe)]. In Scopus, the strategy was modified to search for individual countries, one at a time. Synonymous or previous names of countries were included in the search string, for example, “DR Congo,” “Democratic Republic of Congo,” and “Zaire” for the present-day Democratic Republic of the Congo. Studies published from Sudan before 2011, the year of splitting from South Sudan, were categorized under the former.

### Inclusion and exclusion criteria

Records included in this systematic review met the following criteria: (i) focused on the detection and occurrence of *A. platys* in an African country; (ii) *A. platys* detected in any host species, whether vertebrate or invertebrate; (iii) published in the English language; (iv) full-text availability; (v) published in any year from database inception up to and including December 2024; (vi) cross-sectional or longitudinal observational study design; and (vii) original peer-reviewed journal article, dissertation or thesis, or conference proceeding. Clinical trials that involved observation of natural *A. platys* infections were included. A record was excluded if it was a retracted publication, a book, clinical trial with experimental infection, biography, editorial material, abstract, award grant, dataset, or a duplicate. We also excluded review publications and did not search for gray literature.

### Selection of records and data extraction

Data retrieval, screening, and extraction were performed independently by all three authors (ZTHM, SMM, CB). Any differences in opinion were resolved through discussion to reach a consensus. The selected titles and abstracts were exported to EndNote™ 21 (Philadelphia, PA, USA). Duplicate records were removed using the built-in tools and checked manually at every data processing stage.

Data from eligible studies were extracted in a Microsoft Excel^®^ spreadsheet (version 365, Microsoft Corporation, Redmond, WA, USA), and this included the author names, study title, study and publication years, study design, host species, number of individual hosts studied and positive for the pathogen, country, and detection method(s). In studies that sequenced for full-length or near-full-length *A. platys* genes, we recorded sequence data obtained, for the purpose of phylogenetic analysis. When a record targeted the detection of *A. platys* in different countries, each study was recorded and analyzed separately. Geographical coordinates (altitude, longitude, latitude) of the study areas were searched for on the Latitude and Longitude Finder (https://www.latlong.net/ or https://gps-coordinates.org/).

### Molecular phylogenetics

Phylogenetic relationships were inferred using both the full-length 16S rRNA gene and the *groEL* protein-coding gene sequences obtained from the National Center for Biotechnology Information (NCBI) database. Sequence selection was conducted by searching the NCBI GenBank using the Basic Local Alignment Search Tool (BLAST), whereby accession numbers reported in the published articles were queried, and the resulting matches (at least 99% identity with *A. platys*) were assessed to confirm taxonomic identity and sequence length. From the BLAST outputs, relatively long and near-full-length sequences were considered for inclusion. Moreover, for the 16S rRNA analysis, *A. platys* sequences generated by Khoza et al. [12] by polymerase chain reaction (PCR) and next-generation sequencing (NGS) of the full-length 16S rRNA gene from cattle blood samples in South Africa, under GenBank BioProject accession number PRJNA1031221, were included, together with additional near-full-length sequences retrieved from GenBank (varying from 1313 to 1460 base pairs [bp] for 16S rRNA). For the *groEL* gene, amino acid sequences (varying from 198 to 486 amino acids) were directly downloaded from GenBank; no translation was performed. Corresponding sequences of other *Anaplasma* species as well as outgroup sequences from closely related parasite species were also retrieved from GenBank. The retrieved full-length or near-full-length sequences were trimmed to size equal to the length of the shortest sequence (1313 bp for 16S rRNA and 198 amino acids for *groEL*) for comparative analysis. Multiple sequence alignments for both datasets were carried out using MAFFT (multiple alignment using fast Fourier transform) version 7 [41, 42] and manually inspected in BioEdit version 7.2.5 [43] for accuracy. The best-fit nucleotide substitution model TPM1uf+I+G for the 16S rRNA dataset was identified with jModelTest 2 [44], while the best-fit amino acid substitution model LG+G for the *groEL* dataset was determined using ProtTest 3 [44]. Maximum-likelihood phylogenetic trees for each dataset were constructed in PhyML 3.0 [45], with parameters set according to the selected models. Branch support was evaluated using bootstrap analysis [46] as implemented in PhyML.

### Data analysis

Descriptive statistics analysis in the form of median, mean (continuous variables), numbers, and percentages (categorical variables) was performed to assess the occurrence of *A. platys* across different countries, regions, study years, and host species. Heterogeneity in prevalence estimates was high; therefore, a meta-analysis of the occurrence of *A. platys* across various African countries was not conducted. Data analysis and visualization were conducted using R statistical software version 4.4.1 [47]. Geographical distribution of *A. platys* was visualized by generation of maps using QGIS (Quantum Geographic Information System) software version 3.36.1 [48]. The shapefiles to lay the foundation for the maps were obtained from the open-access DIVA-GIS website (https://www.diva-gis.org/).

## Results

### Search process and results

**Fig. 1:**
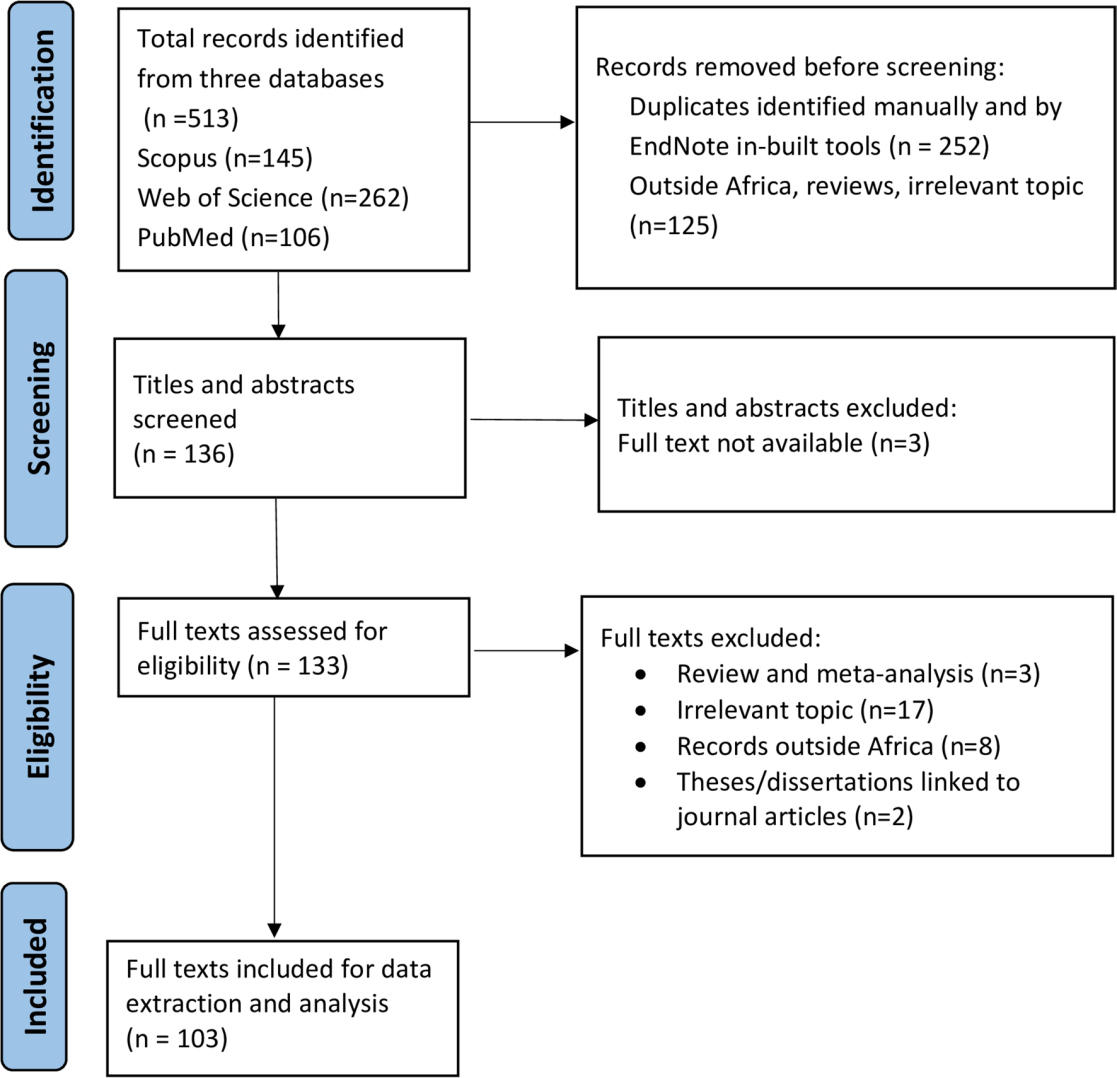
Flow diagram showing the search and selection of studies regarding the detection of *Anaplasma platys* and *A. platys*-like among mammalian and arthropod hosts in Africa. Included studies were of both cross-sectional and longitudinal design, published in English, with the exclusion of reviews or meta-analysis and experimental infections. The Preferred Reporting Items for Systematic reviews and Meta-Analyses guidelines were followed for the search process

**Fig. 2:**
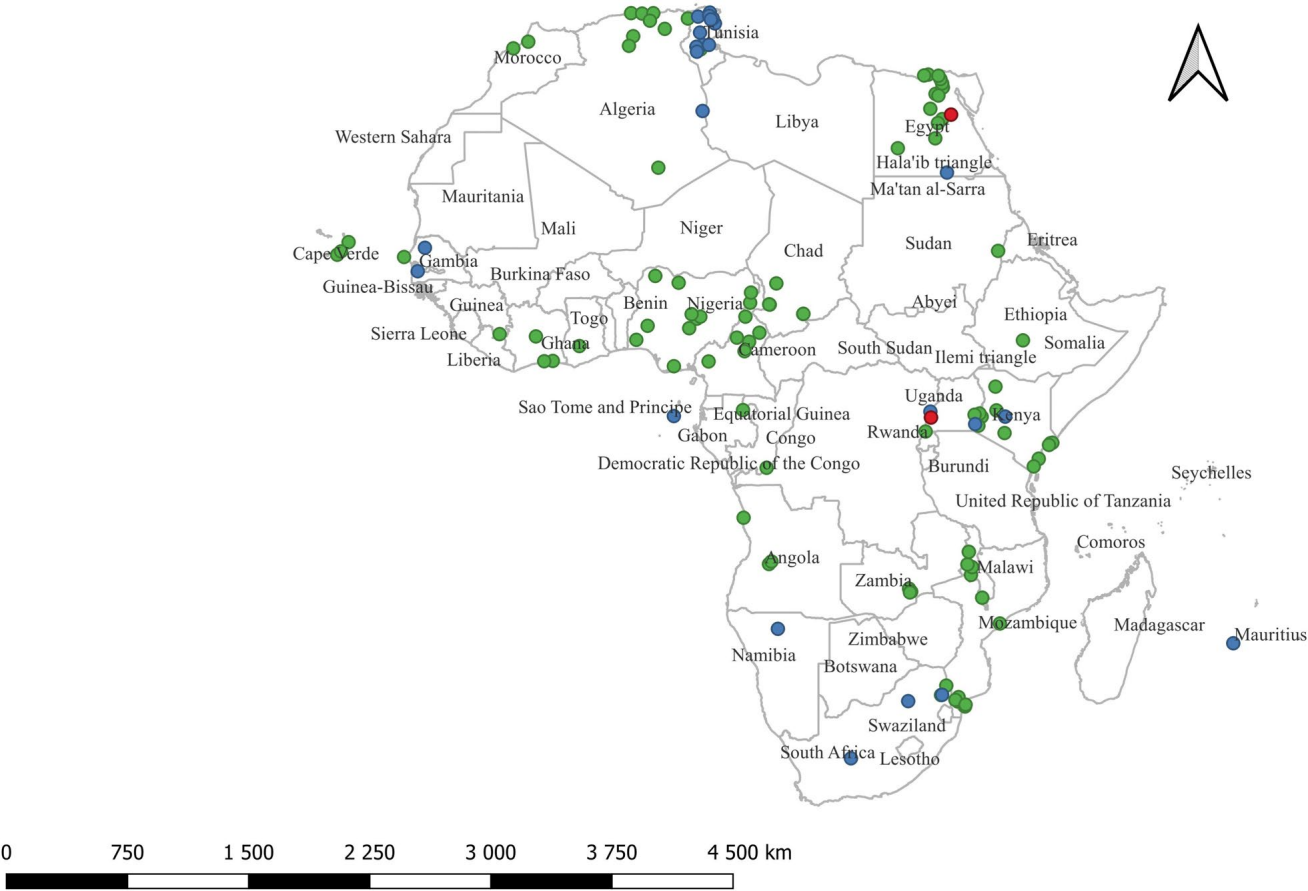
The occurrence of *Anaplasma platys* and *A. platys*-like in various hosts and vectors in African countries (based on studies in the review). *Green dots* indicate *A. platys* species, *blue dots* indicate *A. platys*-like organisms, and *red dots* indicate reports of both organisms

A flow diagram demonstrating selection of records is shown in Fig. 1. The search yielded a total of 513 records: 145 from Scopus, 262 from Web of Science, and 106 from PubMed. After removing 377 records that did not meet the eligibility criteria (duplicates, studies conducted outside of Africa, reviews, and studies irrelevant to the objectives), 136 records were retained for title and abstract screening. Subsequently, three records were excluded because their full texts were not available. The full text of the remaining 131 journal articles and two academic theses (total 133 records) were assessed for eligibility, with the exclusion of 30 records because they were review articles, academic theses linked to journal articles, irrelevant to the study objective, or outside of Africa. No conference proceedings relevant to our objectives were encountered. Therefore, a total of 103 records that met the eligibility criteria were included in the qualitative and quantitative syntheses. Of these records, *A. platys* and/or the closely related *A. platys*-like organisms were detected in only 80 studies (from 79 records). One record reported findings from two countries, Kenya and Côte d'Ivoire, which were recorded as two separate studies. The 80 studies represented 25 countries across the five African regions, as follows: five countries from northern Africa [Algeria (*n* = 7 studies), Egypt (*n* = 10), Morocco (*n* = 2), Sudan (*n* = 1), Tunisia (*n* = 5)]; five from western Africa [Nigeria (*n* = 5), Senegal (*n* = 3), Ghana (*n* = 1), Guinea (*n* = 1), Côte d'Ivoire (*n* = 2)]; five from central Africa [Chad (*n* = 1), Gabon (*n* = 1), Angola (*n* = 3), Cameroon (*n* = 3), Democratic Republic of the Congo (*n* = 1)]; six from eastern Africa [Kenya (*n* = 8) Malawi (*n* = 3), Ethiopia (*n* = 2), Mozambique (*n* = 3), Zambia (*n* = 3), Uganda (*n* = 2)]; two from southern Africa [Namibia (*n* = 1), South Africa (*n* = 8)]; and two island nations [Cape Verde (*n* = 3) and Mauritius (*n* = 1)] (Table 1 and Fig. 2). The study from Sudan was conducted between 1997 and 2000, before independence of South Sudan in 2011. For this reason, together with the fact that the study location was not specified in the article, the article was regarded as a Sudanese study.

**Table 1:** A summary of *Anaplasma platys* and *Anaplasma platys*-like occurrence in various hosts in Africa

Country	Location	*A. platys/A. platys*-like	Sample size	No. positive	Prevalence (%)	Source
Domestic dog						
Egypt	Various	Both	203	7	3.4	[55]
Egypt	Cairo	*A. platys*	124	1	0.8	[83]
Egypt	Cairo	*A. platys*	230	15	6.5	[7]
Egypt	Giza	*A. platys*	110	10	9.1	
Egypt	Qalyubia	*A. platys*	60	2	3.3	
Egypt	Gharbia	*A. platys*	60	3	5.0	
Egypt	Kafr El Sheikh	*A. platys*	40	2	5.0	
Egypt	Alexandria	*A. platys*	70	4	5.7	[84]
Algeria	Tizi Ouzou	*A. platys*	104	12	11.5	[30]
Algeria	Béjaïa	*A. platys*	6	1	16.7	
Algeria	Algiers	*A. platys*	213	31	14.6	[85]
Angola	Luanda	*A. platys*	103	21	20.4	[27]
Ghana	Kumasi	*A. platys*	17	3	17.6	[86]
Senegal	Dakar	*A. platys*	34	1	2.9	[87]
Senegal	Keur Momar Sarr	*A. platys*-like	64	10	15.6	[36]
Zambia	Lusaka, Mazabuka, Monze, Shangombo	*A. platys*	247	4	1.6	[88]
South Africa	Mnisi Community	*A. platys*	56	1	1.8	[89]
South Africa	Mnisi	*A. platys*	10	2	20.0	[39]
Cape Verde	Boa Vista Island	*A. platys*	150	2	1.3	[89]
Malawi	Lilongwe	*A. platys*	197	4	2.0	[90]
Nigeria	Ibadan	*A. platys*	150	1	0.7	[91]
Nigeria	Plateau	*A. platys*	150	9	6.0	[92]
Nigeria	Kwara	*A. platys*	3	1	33.3	
Nigeria	Rivers	*A. platys*	17	2	11.8	
Zambia	Chilanga	*A. platys*	301	20	6.6	[54]
Côte d'Ivoire	Zoukoussi	*A. platys*	18	4	22.2	[17]
Côte d'Ivoire	Irobo	*A. platys*	23	7	30.4	
Côte d'Ivoire	Abidjan	*A. platys*	137	2	1.5	[93]
Kenya	Pate	*A. platys*	10	1	10.0	[17]
Kenya	Mtanga Wanda	*A. platys*	9	2	22.2	
Kenya	Kizingitini	*A. platys*	22	5	22.7	
Kenya	Matondoni	*A. platys*	45	8	17.8	
Kenya	Homa Bay County	*A. platys*	7	4	57.1	[18]
Kenya	Northern	*A. platys*	44	7	15.9	[23]
Cape Verde	Maio Island	*A. platys*	153	53	34.6	[51]
Uganda	Karusandara sub-county	Both	9	9	100.0	[38]
Sudan	Eastern Sudan	*A. platys*	78	19	24.4	[28]
DRC	Kinshasa	*A. platys*	2	1	50.0	[13]
Tunisia	Tunis	*A. platys*	228	10	4.4	[94]
Chad	Mayo-Kebbi	*A. platys*	265	54	20.4	[52]
Chad	Mayo-Kebbi	*A. platys*	185	38	20.5	
Chad	Mayo-Kebbi	*A. platys*	159	19	11.9	
Chad	Mayo-Kebbi	*A. platys*	163	40	24.5	
Chad	Mayo-Kebbi	*A. platys*	125	8	6.4	
Chad	Mayo-Kebbi	*A. platys*	98	7	7.1	
Gabon	Ogooué-Ivindo	*A. platys*	255	3	1.2	[93]
Ethiopia	Gamo Zone	*A. platys*-like	273	25	9.2	[95]
Mauritius	Port Louis	*A. platys*-like	78	12	15.4	[96]
Human						
South Africa	South Africa	*A. platys*-like	1	1	100	[25]
Cattle						
Cameroon	Vina	*A. platys*	396	20	5.1	[97]
Cameroon	Faro et Deo	*A. platys*	198	3	1.5	
Cameroon	Adamaoua	*A. platys*	175	6	3.4	
Cameroon	Mayo–Rey	*A. platys*	310	33	10.6	
Cameroon	Mayo–Tsanaga	*A. platys*	181	8	4.4	
Angola	Huambo	*A. platys*	98	3	3.1	[98]
Angola	Huambo	*A. platys*	88	16	18.2	[99]
Kenya	Kakamega	*A. platys*	272	39	14.3	[100]
Kenya	Bungoma	*A. platys*	99	12	12.1	
Kenya	Busia	*A. platys*	51	6	11.8	
Kenya	Nairobi	*A. platys*	306	13	4.2	[101]
Kenya	Baringo County	*A. platys*	31	4	12.9	[18]
Morocco	Rabat-Sale Kenitra Region	*A. platys*	508	1	0.2	[53]
Zambia	Chilanga	*A. platys*	50	17	34.0	[54]
Nigeria	Borno	*A. platys*	50	16	32.0	[35]
Nigeria	Katsina	*A. platys*	50	2	4.0	
Nigeria	Plateau	*A. platys*	75	34	45.3	
Nigeria	Nasarawa	*A. platys*	50	20	40.0	
Nigeria	Plateau State	*A. platys*	704	27	3.8	[10]
Mozambique	Maputo	*A. platys*	219	36	16.4	[102]
Mozambique	Boane	*A. platys*	50	37	74.0	[34]
Mozambique	Moamba	*A. platys*	50	36	72.0	
Mozambique	Manhiça	*A. platys*	50	38	76.0	
Mozambique	Marracuene	*A. platys*	50	42	84.0	
Egypt	EL-Minya and Assiut	*A. platys*	103	9	8.7	[61]
Egypt	EL-Fayoum	*A. platys*	103	9	8.7	
Egypt	New Valley	*A. platys*	103	8	7.8	
Egypt	Various	Both	88	3	3.4	[55]
Kenya	Kilifi County	*A. platys*	705	13	1.8	[103]
Kenya	Kwale County	*A. platys*	781	26	3.3	
Kenya	Lambwe Valley	*A. platys*-like	680	115	16.9	[104]
Cameroon	Northern Cameroon	*A. platys*	31	13	41.9	[97]
Algeria	Batna	*A. platys*	21	1	4.8	[105]
Senegal	Keur Momar Sarr	*A. platys*-like	15	2	13.3	[36]
Senegal	Sine–Saloum	*A. platys*-like	47	12	25.5	
South Africa	Mnisi	*A. platys*-like	10	7	70.0	[106]
Uganda	Karusandara sub-county	Both	113	13	11.5	[38]
Uganda	Kichwamba sub-county	*A. platys*-like	95	11	11.6	
Tunisia	Bizerte	*A. platys*-like	103	8	7.8	[37]
Tunisia	Nabeul	*A. platys*-like	66	3	4.5	
Tunisia	Ariana	*A. platys*-like	56	2	3.6	
Sheep						
Senegal	Badiouré	*A. platys*	30	1	3.3	[107]
Senegal	Keur Momar Sarr	*A. platys*-like	136	27	19.9	[36]
Egypt	Various	Both	58	1	1.7	[55]
Kenya	Homa Bay County	*A. platys*	21	3	14.3	[18]
Kenya	Mpala Research Center (MRC)	*A. platys*-like	50	7	14.0	[56]
Kenya	Lekiji	*A. platys*-like	84	56	66.7	
Tunisia	Bizerte	*A. platys*-like	85	9	10.6	[37]
Tunisia	Tunis	*A. platys*-like	107	3	2.8	
Tunisia	Beja	*A. platys*-like	66	10	15.2	
Tunisia	Nabeul	*A. platys*-like	56	11	19.6	
Tunisia	Ariana	*A. platys*-like	41	6	14.6	
South Africa	Onderstepoort	*A. platys*-like	1	1	100.0	[49]
Goat						
Kenya	Baringo County	*A. platys*	87	17	19.5	[18]
Kenya	Homa Bay County	*A. platys*	30	2	6.7	
Senegal	Keur Momar Sarr	*A. platys*-like	29	8	27.6	[36]
Tunisia	Bizerte	*A. platys*-like	61	34	55.7	[37]
Tunisia	Beja	*A. platys*-like	48	4	8.3	
Tunisia	Nabeul	*A. platys*-like	37	17	45.9	
Camel						
Egypt	Various	Both	149	1	0.7	[55]
Egypt	Various	Both	149	8	5.4	
Egypt	Abu Simbel	*A. platys*-like	100	29	29.0	[57]
Algeria	Laghouat Province	*A. platys*	80	6	7.5	[108]
Nigeria	Sokoto, northwestern Nigeria	*A. platys*	36	22	61.1	[8]
Tunisia	Gabes	*A. platys*-like	26	4	15.4	[109]
Tunisia	Kebili	*A. platys*-like	250	9	3.6	
Tunisia	Kairouan	*A. platys*-like	40	8	20.0	
Tunisia	Sousse	*A. platys*-like	42	2	4.8	
Tunisia	Bouficha	*A. platys*-like	32	10	31.3	[29]
Tunisia	Sidi Bouzid	*A. platys*-like	155	23	14.8	
Tunisia	Douz	*A. platys*-like	39	7	17.9	
Sable antelope						
Zambia	Lusaka National Park, Chongwe	*A. platys*	47	2	4.3	[59]
Grant’s gazelle						
Kenya	Mpala Research Center	*A. platys*-like	179	58	32.4	[56]
Impala						
South Africa	Kruger National Park	*A. platys*	12	7	58.3	[50]
South Africa	Kruger National Park	*A. platys*-like	21	2	9.5	[33]
African buffalo						
Mozambique	Marromeu District–Sofala Province	*A. platys*	97	7	7.2	[60]
Egypt	AL-Faiyum, AL-Giza, Beni-Suef, AL-Minufia, AL-Beheira, and Matruh	*A. platys*	26	2	7.7	[62]
Egypt	EL-Minia and Assiut	*A. platys*	28	2	7.1	[61]
Egypt	EL-Fayoum	*A. platys*	28	1	3.6	
Egypt	New Valley	*A. platys*	27	1	3.7	
Egypt	Various	Both	26	2	7.7	[55]
South Africa	Kruger National Park	*A. platys*-like	13	1	7.7	[33]
Kudu						
South Africa	Kruger National Park	*A. platys*-like	6	5	83.3	[33]
Zebra						
South Africa	Kruger National Park	*A. platys*-like	6	1	16.7	[33]
Warthog						
South Africa	Kruger National Park	*A. platys*-like	8	1	12.5	[33]
Elephant						
South Africa	Kruger National Park	*A. platys*-like	2	1	50	[33]
Lion						
South Africa	Kruger National Park	*A. platys*-like	6	1	16.7	[33]
Leopard						
South Africa	Kruger National Park	*A. platys*-like	9	1	11.1	[33]
Bat eared fox						
Namibia	Etosha National Park	*A. platys*-like	9	8	88.9	[58]
Brown hyena						
Namibia	Etosha National Park	*A. platys*-like	17	14	82.3	[58]
Spotted hyena						
Namibia	Etosha National Park	*A. platys*-like	19	19	100	[58]

The reviewed studies covered a total of 25 African countries. DRC = Democratic Republic of the Congo

### Detection methods and study years

**Fig. 3:**
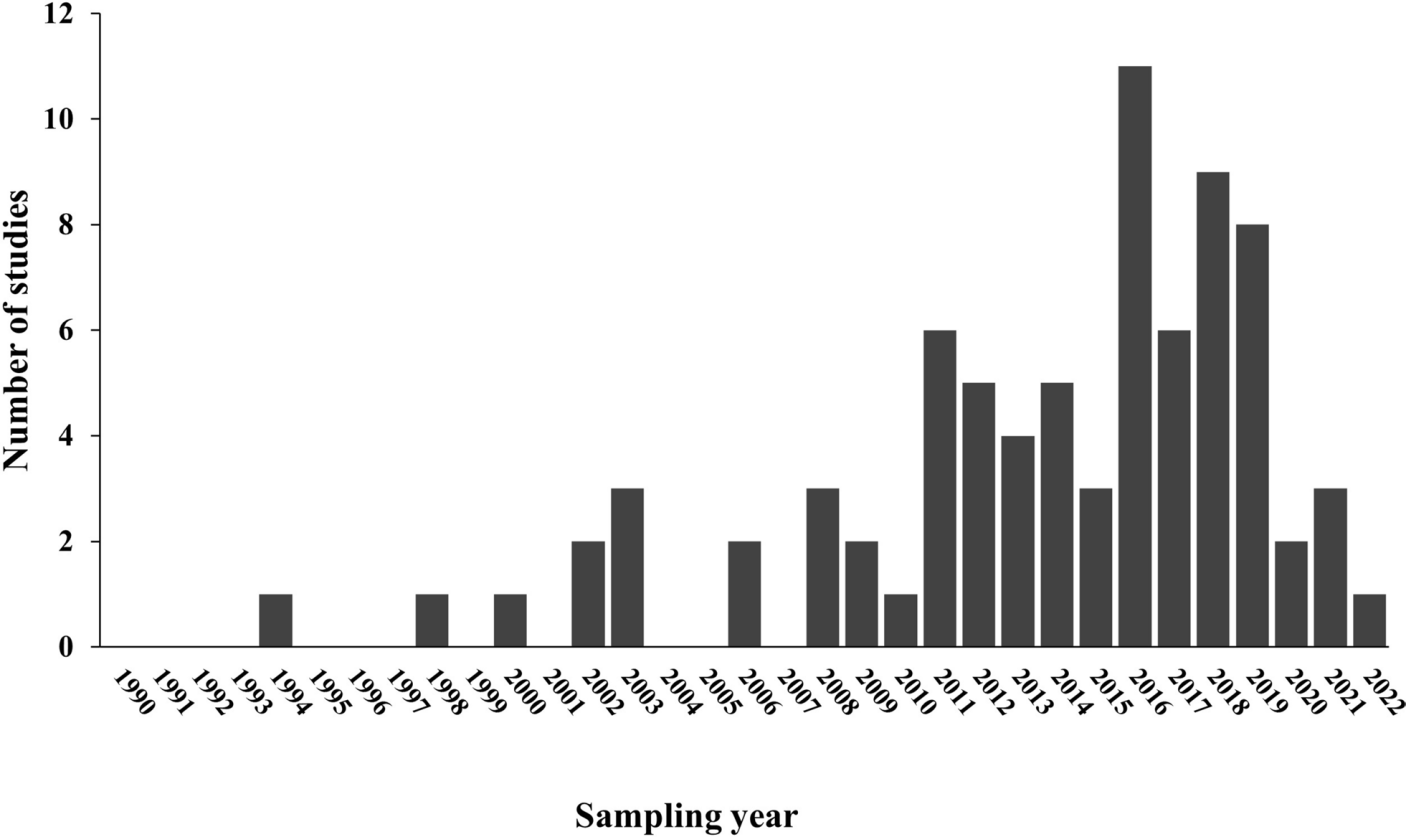
Frequency of detection of *Anaplasma platys* and *A. platys*-like in domestic and wild animals and humans in Africa. The 80 studies were retrieved from Scopus (2004), PubMed (1996), and Web of Science (all databases) (1900). Included studies were published during the period from database inception up to and including December 2024

The 80 studies that detected *A. platys* and/or *A. platys*-like employed a range of laboratory methods. Conventional PCR was the most frequently used method (*n* = 63 studies), followed by quantitative real-time PCR (qPCR) (*n* = 10), reverse line blot (RLB) hybridization (*n* = 7), and PCR–high-resolution melting (PCR-HRM) analysis (*n* = 4). Other methods included microscopy (*n* = 2 studies), restriction fragment length polymorphism (RFLP) (*n* = 1), transmission electron microscopy (TEM) (*n* = 1), the SNAP 4 Dx Plus test (*n* = 1), and a loop-mediated isothermal amplification (LAMP/liquid crystal display [LCD]) assay (*n* = 1). DNA sequences of *A. platys* and *A. platys*-like were confirmed mostly by Sanger sequencing (*n* = 65 studies), and to a lesser extent using NGS (PacBio platform) (*n* = 4 studies). Most studies (*n* = 65/80) targeted the 16S rRNA gene for PCR-based detection, while other gene targets were *groEL* (*n* = 16 studies), 23S rRNA (*n* = 8), *gltA* (*n* = 6), *rpoB* (*n* = 2), *msp2* (*n* = 2), and *msp4* (*n* = 2). The highest detection of *A. platys* was in 2016 (*n* = 11 studies), followed by 2018 (*n* = 9), 2019 (*n* = 8), and 2017 (*n* = 6). Most studies were conducted in and after 2010 (*n* = 64/80, 80%) (Fig. 3).

### Occurrence of *A. platys* and *A. platys*-like DNA in vertebrate hosts and arthropod vectors

Reports of *A. platys* and *A. platys*-like DNA span over two decades, with the earliest detections reported in 1997 in South Africa. The first cases were identified in South Africa: *A. platys*-like DNA in sheep from the Onderstepoort Veterinary Institute [49] and *A. platys* DNA in impala from Kruger National Park [50]. Since these initial reports, *A. platys* and *A. platys*-like DNA have been detected in a wide range of hosts, including domestic animals such as dogs, cattle, sheep, goats, and camels, as well as humans. In wildlife species, it has been reported in African buffalo (*Syncerus caffer*), sable antelope (*Hippotragus niger*), Grant’s gazelle (*Nanger granti*), impala (*Aepyceros melampus*), greater kudu (*Tragelaphus strepsiceros*), plains zebra (*Equus quagga*), warthog (*Phacochoerus africanus*), elephant (*Loxodonta africana*), lion (*Panthera leo*), leopard (*Panthera pardus*), fox (*Vulpes*), and hyena (*Crocuta crocuta* and *Parahyaena brunnea*) (Table 1).

Domestic dogs were the most frequently reported host, in 32 of the 80 studies and across 20 of the 25 countries. Reported prevalence ranged from 0.8% to 100% for *A. platys* and from 9.2% to 15.6% for *A. platys*-like, with sample sizes varying from as few as six to as many as 273 animals. The highest prevalence of *A. platys* was recorded in Cape Verde, where 34.6% of dogs (*n* = 153) on Maio Island were positive [51]. This was followed by Chad, with a prevalence of 24.5% in 163 dogs [52], and other notable hotspots such as Côte d'Ivoire (30.4% in Irobo; [17]), Kenya (22.7% in Kizingitini; [17]), and Sudan (24.4% in eastern Sudan; [28]). Notably, studies from Uganda reported both *A. platys* and *A. platys*-like DNA in 100% of dogs sampled, although this was based on a very small sample size (*n* = 9; [38]). Similarly, relatively high proportions were observed in the Democratic Republic of the Congo (50% in Kinshasa; [13]) and Kenya (57.1% in Homa Bay; [18]), but again these findings were based on small cohorts (< 10 animals), which may not accurately reflect the population prevalence (Table 1).

To date, only a single human case has been documented in Africa, reported in South Africa in 2013, where *A. platys*-like DNA was detected. The case involved an occupationally exposed veterinarian who was co-infected with *A. platys*, *Bartonella henselae*, and *Candidatus* Mycoplasma haematoparvum. The individual reported frequent contact with arthropod vectors and near-daily interaction with persistent bacteremic potential reservoir hosts, notably cats (*B. henselae*) and dogs (*A. platys*, *Ca.* M. haematoparvum) [25].

For other domestic animals, *A. platys* and *A. platys*-like DNA have been widely reported in cattle, sheep, goats, and camels across Africa, with prevalence ranging from very low to very high. In cattle, infections were documented in Cameroon, Angola, Kenya, Morocco, Nigeria, Mozambique, Zambia, and Egypt (Table 1). Prevalence values ranged from as low as 0.2% in Morocco [53] to the highest levels in Mozambique, where several sites exceeded 70%, reaching a maximum of 84% in Marracuene [34]. Similarly high levels were recorded in Nigeria, with 32.0–45.3% of cattle infected in Borno, Nasarawa, and Plateau states [35], and in Zambia’s Chilanga District, where prevalence reached 34% [54]. The consistency of detection across multiple Mozambican sites strongly suggests that *A. platys* is widely established in cattle populations in the country. Both *A. platys* and *A. platys*-like were also confirmed in cattle from Uganda, Egypt, and Kenya, suggesting a broad host expansion beyond canids.

In sheep, *A. platys* and *A. platys*-like were reported in Egypt, Senegal, Kenya, Tunisia, and South Africa, with prevalence varying from 1.7% in Egypt [55] to 100% in a single South African sample studied [49]. Notably high levels were found in Kenya’s Lekiji (66.7%; [56]), Senegal’s Keur Momar Sarr (19.9%; [36]), and Tunisia’s Nabeul (19.6%; [37]). The high prevalence in Lekiji, based on a relatively large sample (*n* = 84), provides strong evidence of established circulation in sheep.

Goats showed prevalence ranging from 8.3% in Beja, Tunisia [37], to high values in Bizerte (55.7%) and Nabeul (45.9%). In Senegal, 27.6% of goats from Keur Momar Sarr were infected [36]). Although some of these reports were based on moderate sample sizes, the repeated detection of *A. platys*-like DNA in Tunisian goats across multiple sites indicates stable presence in small ruminants.

In camels, *A. platys* and *A. platys*-like DNA were reported in Egypt, Algeria, Nigeria, and Tunisia. Prevalence ranged from 0.7% in Egypt [55] to 61.1% in Sokoto, Nigeria [8]. Tunisia also showed notable infection levels, with 31.3% in Bouficha and up to 29% in Abu Simbel, Egypt [57]. These findings demonstrate that camels, often overlooked in vector-borne pathogen studies, may potentially serve as reservoirs for *A. platys*.

In wildlife, *A. platys* and *A. platys*-like DNA were detected across a remarkably wide range of mammalian hosts. Reports include antelopes, Grant’s gazelles, African buffalo, greater kudu, plains zebra, common warthog, elephants, lions, leopards, and several carnivore species. Particularly striking are the high infection levels observed in Namibia’s Etosha National Park, with 100% prevalence in spotted hyenas, 88.9% in bat-eared foxes (*Otocyon megalotis*), and 82.3% in brown hyenas [58]. Similarly, high prevalence was recorded in kudu from South Africa’s Kruger National Park (83.3%) [33]. In the Grant’s gazelles, *A. platys* was found in Kenya’s Mpala Research Center, with prevalence of 32.4% across 179 samples [56]. In Zambia’s Lusaka National Park, infection was detected in 4.3% of 47 sable antelope [59]. In impala, prevalence ranged from 58.3% in early Kruger National Park surveys [49] to 9.5% in more recent studies [33]. Infections were also detected in African buffalo, with detection in Mozambique (7.2%) [60] and Egypt (3.6–7.7%) [55, 61, 62]. Other wildlife species in Kruger National Park, including zebra, warthog, elephant, lion, and leopard, have all yielded positive detections, albeit at lower prevalence (ranging from 7.7% to 50%) [33].

*Anaplasma platys* DNA was also detected in a variety of tick species across various countries (*n* = 12) in Africa. The pathogen was detected in fleas as well. In North Africa, detections were reported in Tunisia, Algeria, Morocco, and Egypt. In West Africa, *A. platys* was identified in ticks from Côte d'Ivoire and Guinea. In Central Africa, detections were reported in Cameroon and the Democratic Republic of the Congo, while East African countries included Kenya and Uganda. In southern Africa, *A. platys* was detected in Malawi and South Africa. (Table 2). Most detections were from *R. sanguineus* sensu lato (*n* = 8 studies), predominantly collected from domestic dogs. In North Africa, *R. sanguineus* s.l. from dogs showed infection rates ranging from 5.3% in Morocco [31] to 75% in Tunisia [63]. In Algeria, prevalence varied between 17.2% in Djelfa and 52% in Bordj Bou Arreridj [64]. In Central Africa, *R. sanguineus* s.l. from cattle in Cameroon showed prevalence of 2.1% [21], while in the Democratic Republic of the Congo, *R. sanguineus* s.l. from dogs showed 4.8% prevalence [13]. Southern African countries, such as Malawi and South Africa, reported *R. sanguineus* s.l. prevalence in dogs ranging from 6.3% to 50% [65, 66]. *Anaplasma platys* DNA was also detected in other tick species. *Rhipicephalus annulatus*, collected mainly from cattle, showed low infection rates in Egypt (0.002–5.6%) [20, 67] and slightly higher prevalence in Algeria (1.4%) [19]. Donkeys in Egypt also harbored *R. annulatus*, with 27.9% testing positive [68]. *Rhipicephalus pulchellus*, *R. pravus*, and *R. evertsi evertsi* collected from livestock in Kenya exhibited infection rates ranging from 6.9% to 26.9% [18]. *Rhipicephalus microplus* from cattle showed 0.3% infection in Cameroon [21] and 11.8% in Guinea [69]. *Rhipicephalus camicasi* from dogs in Kenya demonstrated 3.4% prevalence [17]. Other *Rhipicephalus* species that tested positive in dogs in Côte d'Ivoire included *R. humeralis* (3.5%) and *R. simus* (1.2%) [23]. *Hyalomma* and *Haemaphysalis* species were also implicated. Infection in *Hae. leachi* was reported in Uganda (18.9%) and Côte d'Ivoire (1.2–16.9%) [17, 70], while occurrence in *Hy. excavatum* from cattle was found in Egypt, with prevalence ranging from 2.6% to 16.2% [20]. *Hyalomma dromedarii* from camels in Algeria showed 25% infection [71]. Lastly, fleas (*C. felis felis*, *P. irritans*, and *C. felis canis*) collected from the environment in Ethiopia showed an overall high infection rate of 44% for *A. platys* [22].

**Table 2:** A summary of vector species, host origin or origin, and infection rates of *Anaplasma platys* from African studies

Country	Location	Tick/flea species	Origin of the tick/flea species	Infection rate (%)	Source
Ticks					
Tunisia	Tunisia	*R. sanguineus* s.l.	Dog	75.0	[63]
Cameroon	Dschang, Nkong-Ni, Bafou Kouoptamo, Massangam, and Koutaba	*R. sanguineus* s.l.	Cattle	2.1	[21]
DRC	Kinshasa	*R. sanguineus* s.l.	Dog	4.8	[13]
Malawi	Chikwawa	*R. sanguineus* s.l.	Dog	16.0	[65]
Malawi	Ntchisi	*R. sanguineus* s.l.	Dog	21.4	
Malawi	Kasungu	*R. sanguineus* s.l.	Dog	6.3	
Malawi	Mzimba	*R. sanguineus* s.l.	Dog	50.0	
South Africa	Bushbuckridge	*R. sanguineus* s.l.	Dog	16.4	[66]
Morocco	Casablanca	*R. sanguineus* s.l.	Dog	5.3	[31]
Côte d'Ivoire	Irobo	*R. sanguineus* s.l.	Dogs	4.7	[17]
Algeria	Djelfa	*R. sanguineus* s.l.	Dogs	17.2	[64]
Algeria	Bordj Bou Arreridj	*R. sanguineus* s.l.	Dogs	52.0	
Egypt	El-Faiyum–Oasis	*R. annulatus*	Cattle	4.8	[20]
Egypt	Assiut	*R. annulatus*	Cattle	5.6	
Egypt	Alexandria	*R. annulatus*	Cattle	0.002	[67]
Algeria	Guelma, Annaba, El Tarf	*R. annulatus*	Cattle	1.4	[19]
Kenya	Baringo County	*R. pulchellus*	Livestock	6.9	[18]
Kenya	Homa Bay County	*R. pulchellus*	Livestock	24	
Kenya	Baringo County	*R. pravus*	Livestock	23.1	
Kenya	Homa Bay County	*R. pravus*	Livestock	17.1	
Kenya	Baringo County	*R. evertsi evertsi*	Livestock	23.8	
Kenya	Homa Bay County	*R. evertsi evertsi*	Livestock	26.9	
Guinea	Nzerekore, Faranah, Kankan	*R. microplus*	Cattle	11.8	[69]
Cameroon	Dschang, Nkong-Ni, Bafou, Kouoptamo, Massangam and Koutaba	*R. microplus*	Cattle	0.3	[21]
Kenya	Matondoni	*R. camicasi*	Dog	3.4	[17]
Egypt	Cairo and Beni Suef provinces	*R. annulatus*	Donkey	27.9	[110]
Côte d'Ivoire	Irobo	*R. humeralis*	Dog	3.5	[17]
Côte d'Ivoire	Irobo	*R. simus*	Dog	1.2	
Uganda	Bwindi Impenetrable National Park	*Hae. leachi*	Dog	18.9	[70]
Côte d'Ivoire	Zoukoussi	*Hae. leachi*	Dog	16.90	[17]
Côte d'Ivoire	Irobo	*Hae. leachi*	Dog	1.20	
Egypt	El-Faiyum Oasis	*Hy. excavatum*	Cattle	2.6	[20]
Egypt	Kharga	*Hy. excavatum*	Cattle	16.2	
Algeria	Tamanrasset	*Hy. dromedarii*	Camels	25.0	[71]
Fleas					
Ethiopia	Gambo	*C. felis felis*	Environment	28.0	[22]
Ethiopia	Gambo	*P. irritans*	Environment	67.6	
Ethiopia	Gambo	*C. felis canis*	Environment	25.0	

DRC = Democratic Republic of the Congo

### Phylogenetic relatedness in the 16S rRNA gene

**Fig. 4:**
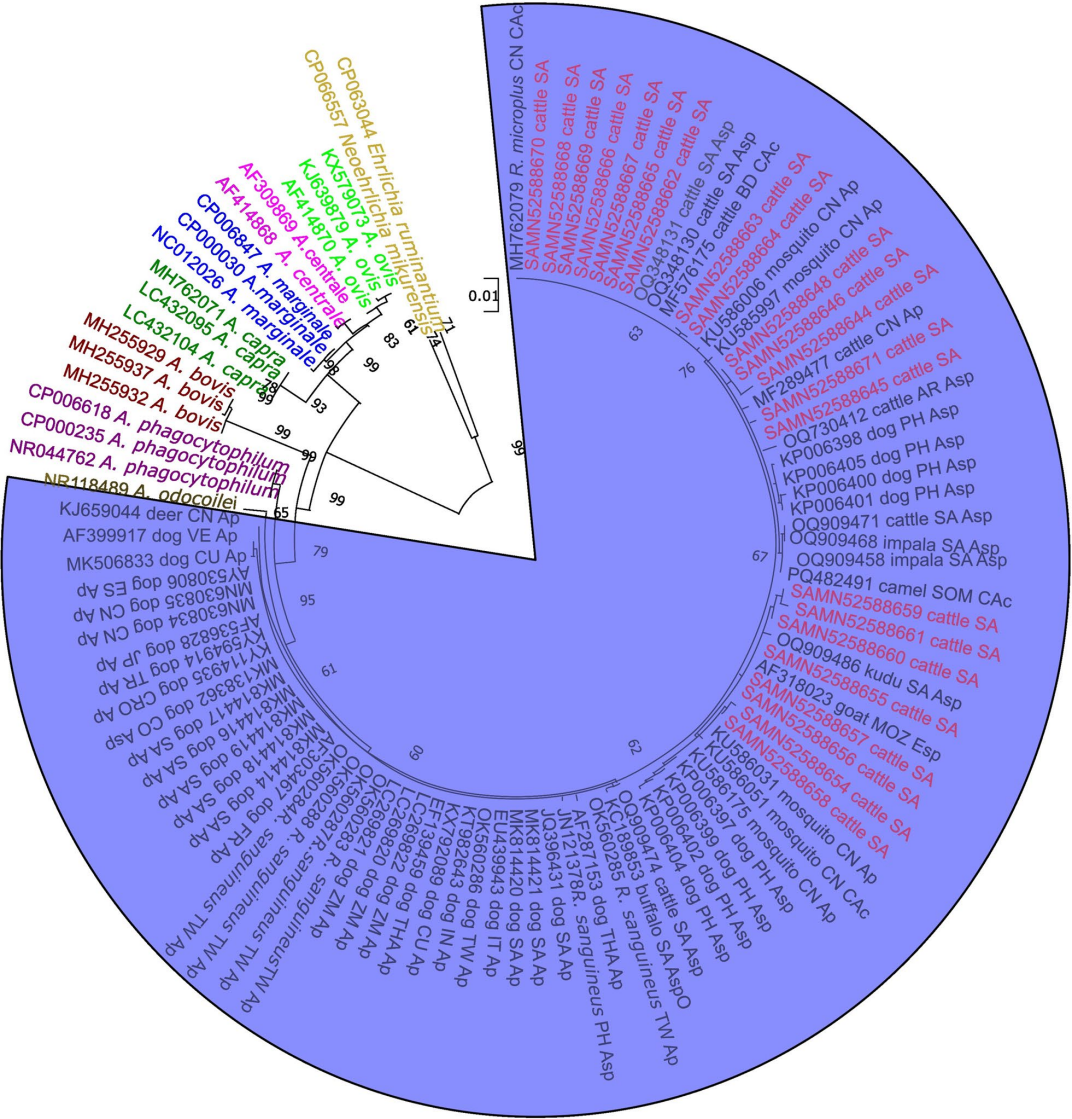
Maximum-likelihood phylogenetic tree based on 16S rRNA sequences showing the relationship of *A. platys* and other *Anaplasma* species. The numbers at the internal nodes represent the percentage of 1000 replicates (bootstrap) for which the same branching patterns were obtained. A total of 1313 nucleotide positions were included in the final dataset. The sequences obtained from Khoza et al. [12] are shown in red within the blue clade. Asp = *Anaplasma* species, Ap = *A. platys*, CAc = *Candidatus* Anaplasma cinensis. Country abbreviations in the tree are as follows: *TW* Taiwan, *IN* India, *CU* Cuba, *CN* China, *THA* Thailand, *ZM* Zambia, *FR* France, *SA* South Africa, *PH* Philippines, *CO* Colombia, *MOZ* Mozambique, *CRO* Croatia, *TR* Turkey, *VE* Venezuela, *SOM* Somalia, *JP* Japan, *ES* Spain, *AR* Argentina, *US* United States. *Rhipicephalus sanguineus* shown in the tree refers to the *R. sanguineus* s.l. species complex

The 16S rRNA maximum-likelihood phylogenetics revealed a distinct monophyletic clustering of *A. platys*, *A. platys*-like, other *Anaplasma* species, and *Candidatus* Anaplasma cinensis derived from diverse hosts and vectors (Fig. 4). Several South African cattle-derived sequences (SAMN52588644–SAMN52588646, SAMN52588648, SAMN52588654–SAMN52588671) from a previous study by Khoza et al. [12] clustered closely with *A. platys*, *A. platys*-like organisms, and other *Anaplasma* species that have been reported from dogs. These sequences exhibited close genetic relationships with *A. platys* isolates from a range of geographical origins, including Cuba (KX792089), Thailand (EF139459), Taiwan (OK560286), Italy (EU439943), India (KT982643), South Africa (MK814420–MK814421, JQ396431, MK814419, MK814416, MK814414, MK814417, MK814418), Zambia (LC269822, LC269820, LC269821), China (MN630835, MN630834), Croatia (KY114935), Turkey (KY594914), France (AF303467), Japan (AF536828), and Spain (AY530806), and additional *A. platys*-like organisms reported from Colombia (MK138362) and the Philippines (KP006397–KP006402, KP006404–KP006405).

In addition, cattle-derived *A. platys* sequences from South Africa (OQ348130–OQ348131 and OQ909471) clustered within the same clade as our cattle-derived sequences, while sequence SAMN52588671 demonstrated higher similarity to an *A. platys* strain detected in cattle from China (MF289477). Sequences derived from impala in South Africa (OQ909458 and OQ909468) were also grouped within the *A. platys* cluster, suggesting a potential overlap of infection among domestic and wild hosts. Notably, mosquito-derived *A. platys* sequences from China formed part of the broader *A. platys* complex, supporting previous observations of possible vector diversification (KU585997, KU586006, and KU586031).

Additional sequences representing *Ca.* A. cinensis clustered as a related lineage, including those originating from mosquitoes in China (KU586051), *R. microplus* ticks (MH762079), and camels in Somalia (PQ482491). The observed clustering pattern is consistent with the highly conserved nature of the 16S rRNA gene, which has been reported to exhibit more than 99.3% sequence identity among distinct *Anaplasma* species [9, 72]. Consequently, while this gene remains a reliable taxonomic marker for species-level identification, it may not adequately resolve intra-species or strain-level diversity.

### Phylogenetic relatedness in the *groEL* gene

**Fig. 5:**
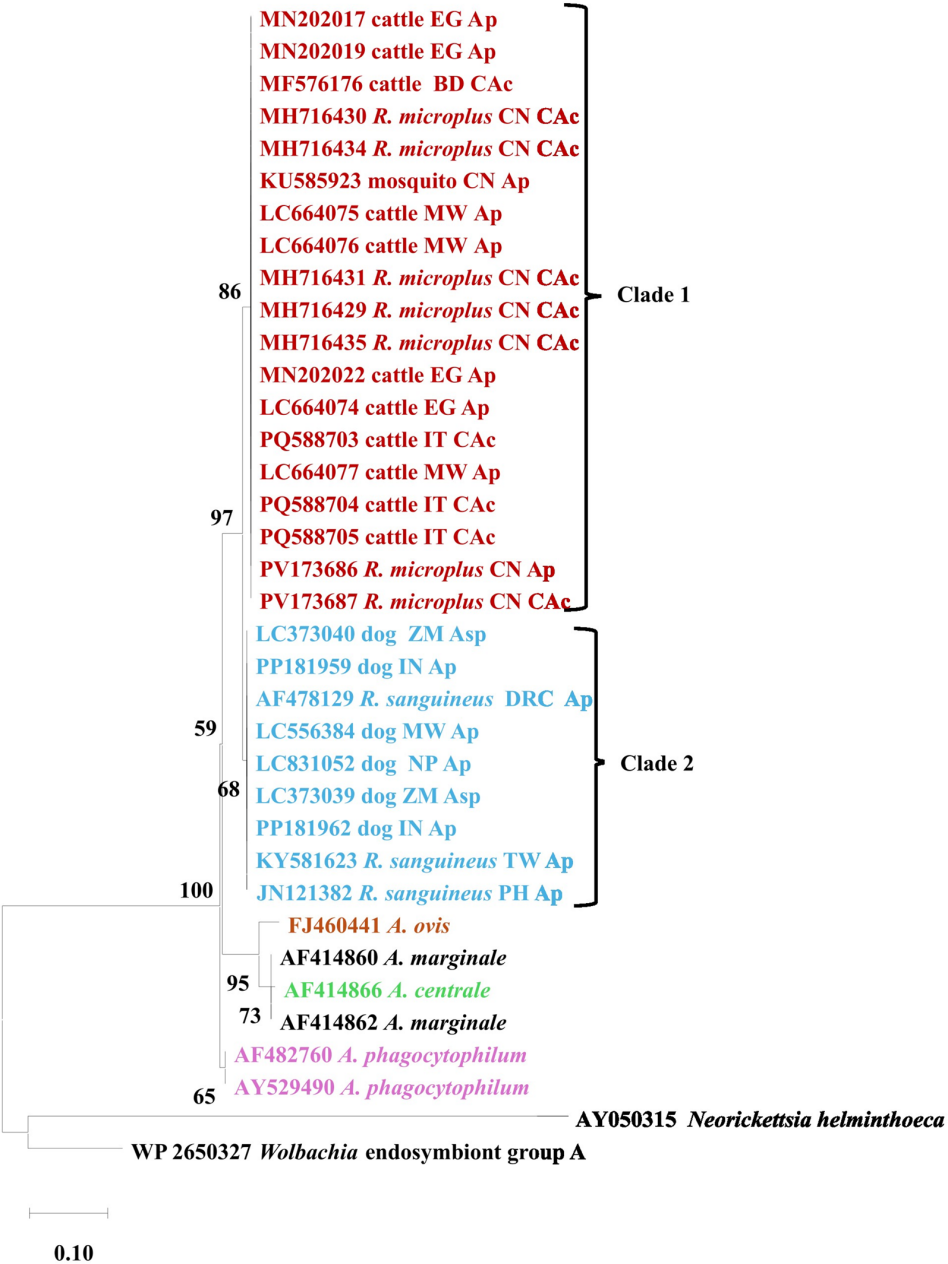
Maximum-likelihood phylogenetic tree based on *GroEL*-deduced amino acid sequences showing the relationship of *A. platys* and other *Anaplasma* species. The numbers at the internal nodes represent the percentage of 1000 replicates (bootstrap) for which the same branching patterns were obtained. A total of 198 positions were included in the final dataset. Asp = *Anaplasma* species, Ap = *A. platys*, CAc = *Candidatus* Anaplasma cinensis. Country abbreviations in the tree are as follows: *EG* Egypt, *BD* Bangladesh, *MW* Malawi, *CN* China, *IT* Italy, *DRC* Democratic Republic of the Congo, *IN* India, *NP* Nepal, *ZM* Zambia, *TW* Taiwan, *PH* Philippines. *Rhipicephalus sanguineus* shown in the tree refers to the *R. sanguineus* s.l. species complex

The *groEL* maximum-likelihood phylogenetic tree (198-bp aligned amino acids) confirmed the 16S rRNA evolutionary relationships among *A. platys*, *A. platys*-like sequences, and related taxa. The analysis resolved two major lineages with varying bootstrap support. The bovine-associated clade (indicated as clade-1) includes *A. platys* sequences from cattle, *R. microplus*, and mosquito-derived sequences, suggesting potential cross-host transmission (Fig. 5). This clade is well supported, with a bootstrap value of 98%. The canine-associated clade (indicated as clade-2) comprises *A. platys* sequences from dogs and associated ticks such as *R. sanguineus*, and this lineage represents the canonical domestic cycle and is supported with a bootstrap value of 68%. Notably, the novel *Ca.* A. cinensis sequences cluster closely with the bovine-associated *A. platys* clade, indicating a possible close evolutionary relationship. The phylogenetic distribution of sequences was not restricted by country (Fig. 5).

## Discussion

This review highlights the wide distribution of *A. platys* and *A. platys*-like organisms across Africa (25 countries), with detection spanning over two decades (1997–2024). Although originally described as a canine-specific pathogen, increasing evidence derived from molecular surveys and case reports suggests a broadened host range and vector spectrum. These findings challenge the long-held assumption that *A. platys* is restricted to dogs and underlines its potential significance in a One Health framework.

Domestic dogs remain the most frequently studied and reported host, with prevalence ranging from low (< 40%) in large-scale Chad surveys to high (> 50%) in smaller studies from Uganda, Kenya, and the Democratic Republic of the Congo. These variations may reflect differences in study design and sample size, but also ecological factors such as host density, vector abundance, or local tick lineages. Importantly, reports from Egypt and Uganda revealed circulation of both *A. platys* and *A. platys*-like organisms in dogs, suggesting possible co-circulation of divergent strains or intra-species genetic diversity. Whether these represent cryptic species or host-adapted lineages remains an unresolved and critical question. This coexistence or co-infection phenomenon is not unheard of in *Anaplasma* species, as several other studies have documented such reports, owing to various vector species that are responsible for horizontal transmission. For example, a study by Qi et al. [73] reported on double, triple, quadruple, and quintuple coexistence/co-infections of various *Anaplasma* species or variants in hedgehogs. It is speculated that the coexistence/co-infections of various *Anaplasma* species in a host may lead to emergence of new variants through genetic recombination [74].

Beyond dogs, high prevalence in cattle, sheep, goats, and camels demonstrates a possible expansion into livestock populations. The particularly high prevalence in Mozambican cattle (> 70%) and Nigerian camels (> 60%) raises the question of whether these animal species may serve as reservoirs or whether they represent spillover hosts in localized transmission systems. Similar complexity emerges from wildlife detections, with DNA evidence reported in antelope, African buffalo, hyenas, and large carnivores. The high occurrence in hyenas and foxes (> 80%) may reflect shared susceptibility among canids but could also indicate adaptation of *A. platys* to wildlife hosts or frequent spillover events in ecosystems where domestic and wild carnivores overlap. Whether these detections correspond to sustained infections capable of continuous transmission, or transient presence of DNA from infected vectors, remains uncertain.

Vector associations further contribute to this ecological complexity. While ticks in the *R. sanguineus* group are widely known as the primary vectors, *A. platys* DNA was also detected in ticks that parasitize both livestock and wildlife, namely *R. annulatus*, *R. pulchellus*, *R. evertsi evertsi*, *R. microplus*, and *Hyalomma* spp. This overlapping host–vector network provides plausible pathways for cross-species transmission and may help explain frequent co-infections with other tick-borne pathogens. The detection of *A. platys* DNA in human, dog, and cat fleas adds another dimension, raising the possibility of alternative transmission routes, including mechanical transmission. This analogy is not without precedent, as hematophagous flies such as *Tabanus*, *Stomoxys*, and mosquitoes have been experimentally shown to transmit *A. marginale* [75, 76]. Although mechanical transmission of *A. platys* has not yet been demonstrated, the parallels suggest that epidemiology may extend well beyond a single tick species.

From a zoonotic perspective, evidence in Africa is limited to a single confirmed human case reported in South Africa in 2013, where a veterinarian who worked on wildlife infested with ticks was infected with a strain closely related to canine *A. platys* [25]. Outside Africa, confirmed infections in Venezuela implicated *R. sanguineus* s.l. [26], and in China, the detection of *A. platys* DNA in mosquitoes [77] points to its underrated zoonotic potential. Given diagnostic limitations and the non-specific clinical presentation of infection, it is highly likely that human cases are underestimated.

The 16S rRNA-based phylogenetic reconstruction of *Anaplasma* sequences obtained from South African cattle [12] revealed that these isolates clustered within the *A. platys*, *A. platys*-like, *Anaplasma* species, and *Ca.* A. cinensis clade, together with sequences previously reported from a variety of hosts and vectors, including dogs, cattle, impala, camels, mosquitoes, and *Rhipicephalus* species. This clustering pattern supports the hypothesis that *A. platys* and *A. platys*-like organisms form a monophyletic lineage with broad host and vector diversity. Similar observations were documented in a review by Rar et al. [78] and by researchers in Italy who have undertaken extensive characterization of *A. platys*-related species. Their studies demonstrated that *A. platys* isolates from dogs, other mammals, and arthropod vectors consistently form a highly conserved monophyletic group with more than 99.3% sequence identity based on the 16S rRNA gene [9, 72].

The present findings extend this understanding by demonstrating that cattle-derived *A. platys*-like sequences from South Africa share close genetic relationships with *A. platys* isolates from diverse geographical regions, including Asia, Europe, and Africa. The detection of these sequences in cattle suggests either cross-species transmission or adaptation of *A. platys*-like strains to bovine hosts. The clustering of some cattle-derived sequences, such as SAMN52588671, with a Chinese cattle-derived *A. platys* strain further supports the potential existence of host-adapted lineages or geographically distinct variants. The co-clustering of *A. platys* sequences from impala and cattle may indicate shared ecological exposure or overlapping transmission cycles between domestic and wild ruminants, highlighting the complexity of *Anaplasma* transmission dynamics in multi-host ecosystems.

Interestingly, mosquito-derived *A. platys* sequences formed part of the broader *A. platys* complex, suggesting that mosquitoes may play a role in the epidemiology of this pathogen. This possibility is particularly compelling in the South African context, where mosquitoes are known to act as mechanical vectors for *A. marginale*. The involvement of mosquitoes could impose additional selective pressures that drive genetic diversification within the *A. platys* complex. However, further experimental and vector competence studies are needed to confirm their role in *A. platys* transmission.

The observation of *Ca.* A. cinensis in a closely related lineage in the phylogenetic tree also warrants attention. Its genetic proximity to *A. platys*-like sequences highlights the challenges associated with differentiating closely related *Anaplasma* taxa using the 16S rRNA gene alone. Given the high degree of conservation of this marker, which can exceed 99.3% identity among distinct *Anaplasma* species, the 16S rRNA gene lacks sufficient discriminatory power for resolving intra-species and strain-level variation. To overcome these limitations, studies should consider incorporating additional genetic markers such as the *groEL*, *gltA*, or *msp4* genes, which have been shown to provide higher phylogenetic resolution among *Anaplasma* species.

On the other hand, the GroEL phylogenetic tree revealed two distinct clades corresponding to different host associations: Clade 1, comprising *A. platys* sequences from cattle, *R. microplus*, and mosquito-derived samples, with good bootstrap support (86%), suggesting possible cross-host transmission, and Clade 2, formed by *A. platys* sequences from dogs and *R. sanguineus*, representing the canonical canine cycle, with relatively good bootstrap support (68%). Interestingly, *Ca.* A. cinensis clustered next to the bovine-associated clade, suggesting a close evolutionary link. The lack of geographical clustering further indicates widespread circulation of both lineages across regions. The revelation of two *A. platys* clusters in the present study contrasts with a previous review [78], which showed only one *groEL* cluster of the pathogen, which was monophyletic with four sub-clusters of *A. platys*-like. It seems that *A. platys* is more diverse than currently documented, and the extent of diversity will become more elaborate with more sequencing data from Africa.

These findings highlight important dynamics in host–pathogen biology. While ancestral *A. platys* exhibit a platelet tropism in dogs, ruminant-associated *A. platys*-like organisms are predominantly neutrophilic [9, 72]. Despite this divergent cell preference, ruminant strains consistently nest within the *A. platys* complex, albeit on branches distinct from the canine lineage. This inconsistency raises questions regarding the evolutionary drivers of divergence, potentially host-specific immune pressures, variation in vector competence among *R. sanguineus* s.l. lineages, and genetic recombination during co-infection within vectors. Collectively, these observations reinforce the concept of *A. platys* as a species complex comprising multiple host-adapted ecotypes.

This interpretation aligns with the evolving phylogenetic framework proposed by Dumler et al. [3] and later expanded to include emerging taxa such as *Ca.* A. cinensis, which exhibits close similarities to both *A. platys* and *A. phagocytophilum*. Analyses of 16S rRNA and *groEL* sequences corroborate this relationship, though the continued “Candidatus” status underscores the incomplete characterization and unresolved taxonomy within the *A. platys* complex. Whether these divergent lineages represent novel species or host-adapted variants remains an open question with significant implications for systematics, vector ecology, and diagnostic development. Additional sequence data from a range of target genes are needed to clarify the phylogenetics of *A. platys*. The *gltA* gene, for example, was previously utilized to characterize the Asian strains with the demonstration of two genetically different lineages of *A. platys*, which are associated with different tick species and mosquitoes, and with one of the lineages more closely related to *A. phagocytophilum* [79] There are scant data about *A. platys gltA* sequences from Africa, and pathogen diversity in this regard remains unknown.

Despite its broad host and vector associations, *A. platys* remains profoundly understudied, and when reported, it is either incidental or a mere co-infection with other *Anaplasma* species. There is still poor understanding of the biological transmission cycle, definitive reservoir hosts involved, and the clinical consequences of infection in livestock, wildlife, and humans. The mild or subclinical presentation of *A. platys* infections in dogs likely contributes to under-detection, while the cyclical nature of rickettsiaemia can result in false negatives when only single-time-point sampling is done [1]. This necessitates sensitive molecular diagnostics and repeated sampling to capture true prevalence and to detect co-infections. A few studies have demonstrated the vectorial capacity of ticks in the *R. sanguineus* group as vectors of *A. platys* [15, 16]. Transstadial and transovarial transmission by *R. sanguineus* sensu stricto Latrielle, 1806 (temperate regions of South and North America, Europe) that originated from eastern Arizona (USA) was demonstrated in white rabbits in New Zealand [16], while another study demonstrated transstadial transmission by *R. sanguineus* s.l. (actual taxonomic status not ascertained) from shelter dogs in Turkey [15]. However, most reviewed studies did not identify beyond *R. sanguineus* s.l., referring to the taxon *R. sanguineus* as a single species, without acknowledging the recent taxonomic status that recognizes the *R. sanguineus* complex as a composition of several distinct lineages or species. The studies therefore oversimplified assumptions about pathogen transmission by ticks in this species complex. Sixteen species constitute the *R. sanguineus* complex [80, 81], and these are morphologically and phylogenetically related to *R. sanguineus* s.s. [81]. Of the 16 species, nine are distributed in Africa: *Rhipicephalus linnaei* Audouin, 1826, which has been designated as part of the “tropical lineage” of *R. sanguineus* s.l. [82], *R. camicasi* Morel, Mouchet and Rodhain, 1976 (northeastern Africa), *R. sulcatus* Neumann, 1908 (Afrotropical distribution: western, central, eastern, southern Africa), *R. turanicus* Pomerantzev, 1936 (mainly Palearctic region of Europe, Middle East, Asia, and some parts of Africa), *R. guilhoni* Morel and Vassilades, 1963 (across the Africa band that borders the southern Sahara, from Senegal and Mauritania to South Sudan and Ethiopia), *R. pusillus* Gil Collado, 1936 (Palearctic region), *R. leporis* Pomerantzev, 1946 (Central Asia, parts of Africa, Middle East), *R. moucheti* Morel, 1965 (West Africa), and *R. afranicus* Bakkes, 2020 (across Africa: formerly referred to as *R. turanicus* in Africa). The term *R. sanguineus* s.l. is reserved for ticks in this group that have not been properly designated. Although *R. linnaei* predominates in sub-Saharan Africa, and African detections of *R. sanguineus* s.l. ticks are likely linked to this tick, the direct epidemiological relevance of the distinct lineages and species of the *R. sanguineus* complex cannot be overlooked. Further investigations are needed to explore the vectorial capacity for *A. platys* by other tick species in the *R. sanguineus* complex in different geographical locations. Given the close morphological and genetic relations, investigations of the tick species responsible for transmission of *A. platys* should integrate traditional morphological and molecular techniques and distribution maps. In addition to this taxonomic uncertainty, most studies did not report how they excluded the possibility of detecting *A. platys* DNA from residual blood meals in ticks or fleas. While collection and extraction procedures were described, they generally did not specify whether measures such as blood meal digestion status or host–origin controls were considered. This limits the ability to definitively attribute *A. platys* detections to true vector infection rather than incidental acquisition from a recent blood meal.

## Conclusions

*Anaplasma platys* is not confined to dogs but circulates widely among livestock, wildlife, and possibly humans, with evidence of multiple tick and non-tick vectors facilitating their persistence. Although zoonotic cases remain rare, occupational exposures and ecological overlap with humans highlight potential public health relevance. This review identifies various research gaps in Africa, and addressing these is critical. Key priorities include (i) defining the biological transmission cycle and confirming vector competence beyond *R. sanguineus* s.l., (ii) clarifying whether livestock and wildlife serve as incidental hosts or true reservoirs, (iii) resolving the taxonomic status of *A. platys*-like organisms with distinct cell tropisms, and (iv) assessing the clinical impact of infection across animal and human hosts. Answering these questions within a One Health framework that integrates veterinary, wildlife, and public health perspectives is essential for unraveling the epidemiology, ecology, and zoonotic potential of *A. platys* in Africa.

## Data Availability

All data used and analyzed in this study are represented in Tables 1 and 2. All data used and analyzed in this study are represented in Tables 1 and Table 2. Genetic sequence data are registered in GenBank BioProject, accession number PRJNA1031221.
